# Supplementary figures and images for: Identifying healthcare experiences associated with perceptions of racial/ethnic discrimination among veterans with pain: A cross-sectional mixed methods survey
Source: PLoS One. 2020 Sep 3;15(9):e0237650. doi: 10.1371/journal.pone.0237650 (PMC7470400; doi:10.1371/journal.pone.0237650)

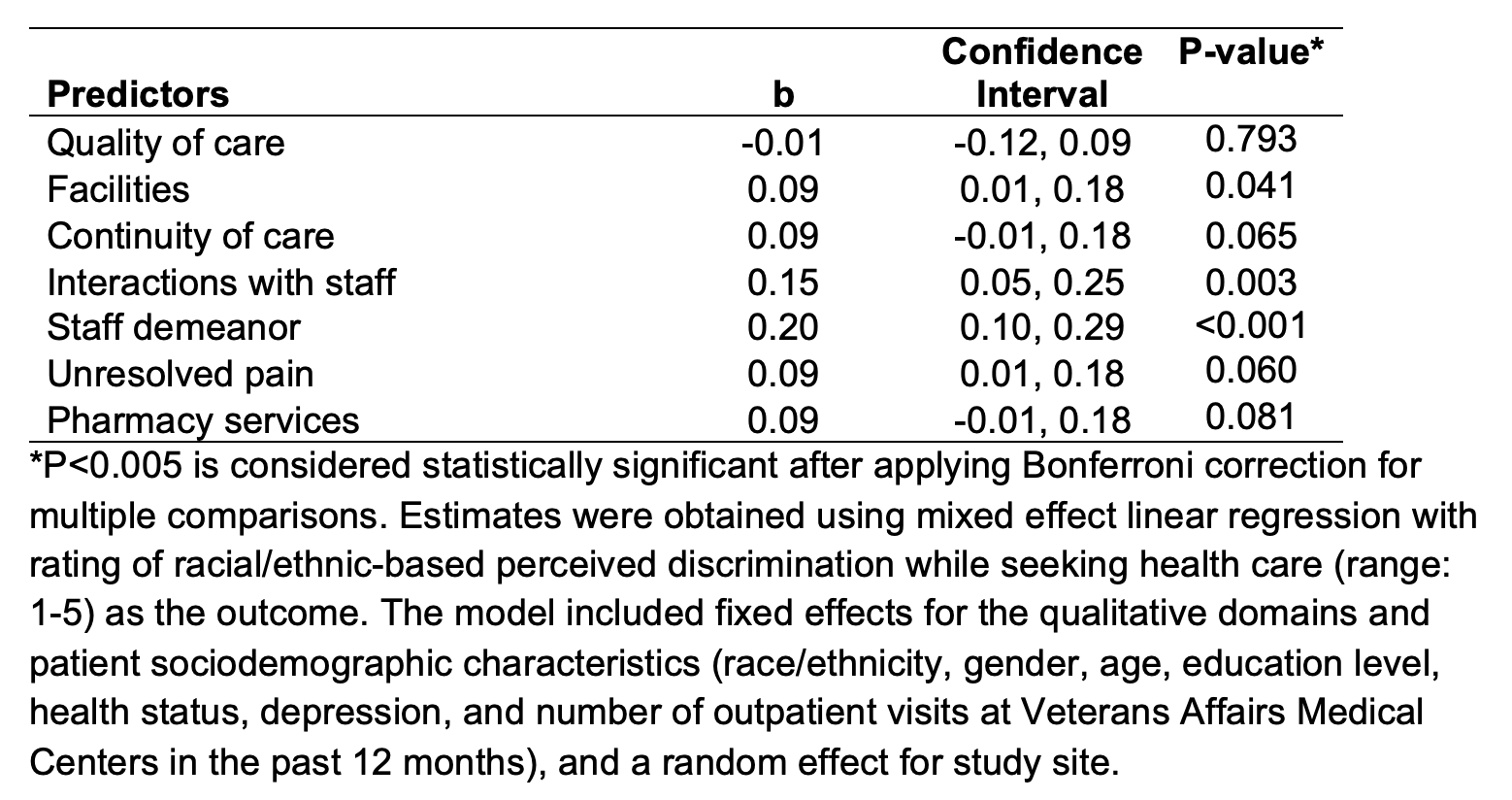

Supplement: S1 Table — *P<0.005 is considered statistically significant after applying Bonferroni correction for multiple comparisons. Estimates were obtained using mixed effect linear regression with rating of racial/ethnic-based perceived discrimination while seeking health care (range: 1–5) as the outcome. The model included fixed effects for the qualitative domains and patient sociodemographic characteristics (race/ethnicity, gender, age, education level, health status, depression, and number of outpatient visits at Veterans Affairs Medical Centers in the past 12 months), and a random effect for study site. (TIFF) [file pone.0237650.s001.tiff]
